# Where should I publish my scientific article? Insights from the editors of Critical Care Science and Critical Care Resuscitation

**DOI:** 10.62675/2965-2774.20250188

**Published:** 2025-07-15

**Authors:** Jorge Ibrain Figueira Salluh, Pedro Henrique Rigotti Soares, Elisa Estenssoro, Ary Serpa, Rinaldo Bellomo, Antonio Paulo Nassar

**Affiliations:** 1 Instituto D’Or de Pesquisa e Ensino Rio de Janeiro RJ Brazil Instituto D’Or de Pesquisa e Ensino - Rio de Janeiro (RJ), Brazil.; 2 Universidade Federal do Rio de Janeiro Rio de Janeiro RJ Brazil Postgraduate Program of Internal Medicine, Universidade Federal do Rio de Janeiro - Rio de Janeiro (RJ), Brazil.; 3 Hospital Nossa Senhora da Conceição Intensive Care Unit Porto Alegre RS Brazil Intensive Care Unit, Hospital Nossa Senhora da Conceição - Porto Alegre (RS), Brazil.; 4 Universidade Federal do Rio Grande do Sul Hospital de Clínicas de Porto Alegre Intensive Care Unit Porto Alegre RS Brazil Intensive Care Unit, Hospital de Clínicas de Porto Alegre, Universidade Federal do Rio Grande do Sul - Porto Alegre (RS), Brazil.; 5 UNISINOS School of Health Department of Internal Medicine Porto Alegre RS Brazil Department of Internal Medicine, School of Health, UNISINOS - Porto Alegre (RS), Brazil.; 6 Escuela de Gobierno en Salud Ministerio de Salud Buenos Aires Argentina Escuela de Gobierno en Salud, Ministerio de Salud - Buenos Aires, Argentina.; 7 Universidad Nacional de La Plata Facultad de Ciencias Médicas Buenos Aires Argentina Facultad de Ciencias Médicas, Universidad Nacional de La Plata - Buenos Aires, Argentina.; 8 Hospital Interzonal de Agudos General San Martín de La Plata Intensive Care Unit Buenos Aires Argentina Intensive Care Unit, Consultant, Hospital Interzonal de Agudos General San Martín de La Plata - Buenos Aires, Argentina.; 9 Monash University School of Public Health and Preventive Medicine Australian and New Zealand Intensive Care Research Centre Melbourne Australia Australian and New Zealand Intensive Care Research Centre, School of Public Health and Preventive Medicine, Monash University - Melbourne, Australia.; 10 Austin Hospital Department of Intensive Care Melbourne Australia Department of Intensive Care, Austin Hospital - Melbourne, Australia; 11 University of Melbourne Melbourne Medical School Department of Critical Care Melbourne Australia Department of Critical Care, Melbourne Medical School, University of Melbourne, Austin Hospital - Melbourne, Australia.; 12 Hospital Israelita Albert Einstein Department of Critical Care Medicine São Paulo SP Brazil Department of Critical Care Medicine, Hospital Israelita Albert Einstein - São Paulo (SP), Brazil.; 13 Austin Hospital Data Analytics Research and Evaluation (DARE) Centre Melbourne Australia Data Analytics Research and Evaluation (DARE) Centre, Austin Hospital - Melbourne, Australia.; 14 A.C. Camargo Cancer Center Intensive Care Unit São Paulo SP Brazil Intensive Care Unit, A.C. Camargo Cancer Center - São Paulo (SP), Brazil.; 15 Instituto D’Or de Pesquisa e Ensino São Paulo SP Brazil Instituto D’Or de Pesquisa e Ensino - São Paulo (SP), Brazil.

## INTRODUCTION

When a critical care research project is initiated, investigators usually think of the final products and the peer-reviewed scientific publication of the findings. This is a fundamental part of the academic process and is not only a marker of success but an essential step to disseminate knowledge and ultimately improve the care and outcomes of patients. Thus, scientific publications are recognized as a trustworthy means to contribute to medical education, scientific debate, and clinical practice. In the last decades, we have observed significant growth in scientific publications, and the immense number of existing journals also mirrors this. If we consider only PubMed®-indexed journals, there are currently more than 5,000 biomedical journals, and over 100 cover acute and critical care. Although this may sound like good news, it has become complex for researchers to choose where to submit their manuscripts.

### What should an author look for before submitting an article?

When preparing to write a manuscript, authors must consider several important factors that will enhance the likelihood of their article being read and maintain its academic integrity. These factors can be categorized into three main areas: suitability, credibility, and visibility.

### Suitability

One of the most crucial steps for authors is to ensure that the journal's scope aligns with their research topic and methodological approach. This alignment, whether the journal is general, focused on critical care, or specialized in a particular subdomain, significantly impacts the relevance and potential impact of the submission.

### Credibility

Authors should check if the journal is indexed in prestigious databases. The Journal Impact Factor, despite its historical role as a fundamental for journal prestige and research influence, has increasingly been revisited for methodological limitations and misapplication in individual-level evaluation. As a means of citation count, the Journal Impact Factor is susceptible to strategies that inflate citations (e.g., self-citation, preferential publication of review articles). It fails to account for the distribution of citations within journals, thereby misrepresenting the impact of most published articles. Emerging alternatives – such as the Eigenfactor Score, Article Influence Score, and SCImago Journal Rank – offer more varied analysis, incorporating citation context and cross-disciplinary comparability. Moreover, article-level metrics (ALMs) and altimetrics enable research assessment beyond academia, including public engagement and policy influence.^(1)^ In an increasingly complex and data-rich scholarly system, continued reliance on the Journal Impact Factor as a singular evaluative standard is reductive and potentially detrimental to advancing open, interdisciplinary, and socially relevant science.^(2)^ The expertise of the editorial board and the rigor of the peer review process – ensuring fairness, transparency, and clarity – are also critical components of a credible journal. The editorial process during manuscript submission should be transparent, enabling authors to follow each stage clearly and confidently.^(2)^

### Visibility

Understanding the journal's readership – whether it primarily targets clinicians, researchers, or other stakeholders – can guide authors in selecting a platform that maximizes the dissemination of their findings. Furthermore, authors should consider the journal's geographical scope (regional, international, or multidisciplinary) and its open access policy, determining if it provides free author access or charges publication fees. The duration of the review and decision-making process is a practical consideration, as longer timelines may delay the dissemination of vital research. But with the proper visibility, the reach of your work is greater.

### Barriers for publication

There are at least four main barriers to publication: the differences between native authors’ language and the official journal language, unaware editors’ and reviewers’ biases, the quality of the peer-review process, and the costs of publication.

The most prestigious biomedical (and critical care) journals are all published in English. Writing in English can be challenging for many non-native English speaker researchers, particularly for authors from Low- and Middle-income Countries (LMICs). Language barriers may lead to misinterpretation and prejudice during the peer review process.^(3)^ Although artificial intelligence tools have helped to overcome language barriers, language biases operate not only in human reviewers but in GPT detectors, which raises concern about the equity of the reviewing processes. Sometimes, the leading journals may seem unreachable to authors from LMICs. At the core of this is the presumption that studies from LMICs might be perceived to be of lesser quality by default. Another cause of bias is an alleged lack of generalizability of the knowledge generated outside high-income regions, but this might reflect a colonialist perspective.^(4)^ Indeed, high retraction rates, the possibility of plagiarism, and research misbehavior regarding credibility have been especially highlighted in some LMICs with high publication rates. However, these considerations should not be automatically translated to other LMICs.

Financial Costs: although open access offers several advantages, such as greater visibility, its article-processing charges impact especially LMIC researchers. The lack of funding for researchers and the lower purchasing power of LMICs limit the number of researchers who can submit their research.^(5)^ Additionally, some journals do not offer discounts for middle-income authors; yet, to be fair, most journals consider discounts after a formal request. However, this is only done after a few email exchanges, which can take weeks. Open-access publications are usually free for low-income countries, but their scientific production is extremely low.^(6)^

### Critical Care Science and Critical Care Resuscitation Journals’ visions

Despite being originally from distinct geo-economic realities (Brazil and Australia-New Zealand), Critical Care Science and Critical Care Resuscitation share similar missions and values. It is the journal editors’ understanding that a transparent and rigorous editorial process is only meaningful when a partnership with authors and readers exists. Both journals are open to submissions in all areas of critical care. In the end, good science is what matters. Backed by strong national medical societies, Critical Care Science and Critical Care Resuscitation warrant credibility and a large audience represented by a growing international community of healthcare professionals practicing critical care. Both journals have strategies to self-fund and not charge authors or readers, translating the published manuscripts into a proper full-access system, which will further increase the scope of the audience. Moreover, a fast, transparent, and fair peer-review process is a strong commitment supported by the diverse international boards. Finally, as high-quality research is our first concern as opposed to a simple metrics-based prioritization, we hope to attract and publish articles from leading international experts that will increase current knowledge and improve care delivery for the critically ill.

## DECLARATION OF GENERATIVE ARTIFICIAL INTELLIGENCE AND ARTIFICIAL INTELLIGENCE-ASSISTED TECHNOLOGIES IN THE WRITING PROCESS

While preparing this work, the author used Grammarly to revise the English language. After using this tool/service, the authors reviewed and edited the content as needed and took full responsibility for the content of the publication
